# Effect of acetic acid on telomerase activity in premalignant and malignant cervical lesions

**DOI:** 10.1038/sj.bjc.6690674

**Published:** 1999-09

**Authors:** I H Güllü, M Kurdoğlu

**Affiliations:** Institute of Oncology, Hacettepe University, Sihhiye, Ankara, 06100, Turkey


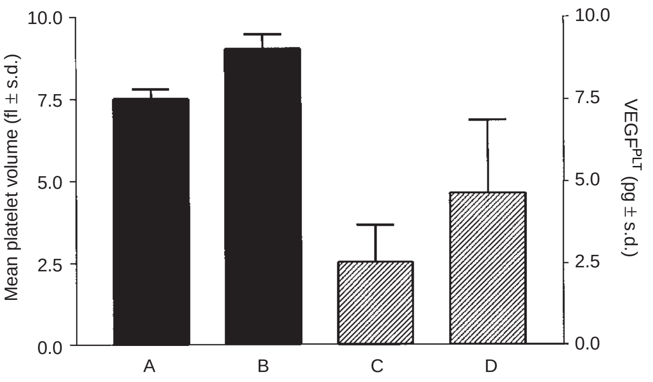

**Figure 1** Mean platelet volumes (MPV, black columns) and corresponding VEGF$^{PLT}$ (hatched columns) in a patient undergoing myeloablative chemotherapy. MPV before the platelet nadir (A) were lower than those thereafter (B, $P = 0.001$). Also, the VEGF$^{PLT}$ are lower before the platelet nadir (C) than thereafter (D). VEGF$^{PLT}$ correlated to the MPV ($r = 0.59$)

increase in the VEGF$^{PLT}$ value from 2.1 ± 1.0 pg during the time of remission to 6.75 pg at the time of clinical relapse. This increment in VEGF$^{PLT}$ could be due to a release of VEGF from a fast-growing tumour mass and/or from tumour-activated platelets (Pinedo et al, 1998). We did indeed find tumour cells to induce platelet activation and release of VEGF from activated platelets (manuscript in preparation).

In summary, we agree with Vermeulen and colleagues that VEGF levels in serum may be a useful tumour marker that relates to the disease stage, tumour progression and tumour-induced platelet activation. To correctly interpret serum VEGF levels, however, blood platelet counts and the platelet size must be taken into consideration.

*E Gunsilius and G Gastl*
*Division of Hematology and Oncology, University Hospital, Anichstr. 35, 6020 Innsbruck, Austria*

#### REFERENCES

Vermeulen PB, Salven P, Benoy I, Gasparini G and Dirix LY (1999) Blood platelets and serum VEGF in cancer patients [letter]. *Br J Cancer* **79**: 370–373

Kraft A, Weindel K, Ochs A, Marth C, Zmija J, Schumacher P, Unger C, Marme D and Gastl G (1999) Vascular endothelial growth factor in the sera and effusions of patients with malignant and nonmalignant disease. *Cancer* **85**: 178–187

Pinedo HM, Verheul HM, D'Amato RJ and Folkman J (1998) Involvement of platelets in tumour angiogenesis? *Lancet* **352**: 1775–1777

### Effect of acetic acid on telomerase activity in premalignant and malignant cervical lesions

### Sir

We read with great interest the report by Mutirangura et al (1998) on defining a correlation between telomerase activity and human papillomavirus (HPV) in normal control tissue and in benign, premalignant and malignant cervical lesions.

We are convinced of the conclusion that there may be two roles of telomerase in the cervix (the first one would present in benign lesions; the second is associated with cancer development and activated during the late stage of multistep carcinogenesis). However, we have doubt about the low percentages of telomerase activity reported for high-grade squamous intraepithelial lesions (SILs) as 40%, and probably for others, especially after reading the article by Changchien et al (1998). The studies of many authors revealed that 25–58% of high-grade SILs exhibited telomerase activity (Kyo et al, 1997; Pao et al, 1997; Zheng et al, 1997). However, Changchien et al reported a relative high percentage of telomerase activity (77.1%) in high-grade SILs. The large discrepancy between the results of previous studies and the results of them were explained by the methods of cervical tissue collection. They claimed that they increased the detection rate of telomerase activity by making the tissues submitted for telomerase assay free of acetic acid.

It is well known that swabbing of cervix with 3–5% acetic acid is a crucial step when colposcopy is performed. Since some of the samples of Mutirangura et al were also obtained by this way, those samples could have been affected by acetic acid. Due to the reason that pH of 5% acetic acid is too low and exposure time is too long, telomerase protein is probably irreversibly denatured by this process (Lodish et al, 1995).

We would like to stress that if 'acetic acid' factor is taken into consideration in such studies, much higher telomerase detection rates and eventually much more accurate results of it are possible to be obtained.

*IH Güllü and M Kurdoğlu*
*Institute of Oncology, Hacettepe University, Sihhiye 06100, Ankara, Turkey.*

#### REFERENCES

Chang Chien C, Lin H, Leung SW, Hsu C and Cho C (1998) Effect of acetic acid on telomerase activity in cervical intraepithelial neoplasia. *Gynecol Oncol* **71**: 99–103

Kyo S, Takakura M, Ishikawa H, Sasagawa T, Satake S, Tateno M and Inouc M (1997) Application of telomerase assay for the screening of cervical lesions. *Cancer Res* **57**: 1863–1867

Lodish H, Baltimore D, Berk A, Zipursky Lawrence S, Matsudira P and Darnell J (1995) A protein can be unfolded by heat, extreme pH and certain chemicals in molecular cellular biology. In: *Protein Structure and Function*, 3rd edn, pp. 73–75. Scientific American Books

Mutirangura A, Sriuranpong V, Termrunggraunglert W, Tresukosol D, Lertsaguansinchai P, Voravud N and Niruthisard S (1998) Telomerase activity and human papillomavirus in malignant, premalignant and benign cervical lesions. *Br J Cancer* **78**: 933–939

Pao CC, Tseng CJ, Lin CY, Yang FP, Hor JJ, Yao DS and Hsueh S (1997) Differential expression of telomerase activity in human cervical cancer and cervical intraepithelial neoplasia lesions. *J Clin Oncol* **15**: 1932–1937

Zheng PS, Iwasawa T, Yokoyama M, Nakao Y, Pater A and Sugimori H (1997) Telomerase activation in in vitro and in vivo cervical carcinogenesis. *Gynecol Oncol* **66**: 222–226

 *© 1999 Cancer Research Campaign*

